# Impaired awareness of hypoglycemia is associated with progressive loss of heart rate variability in patients with type 1 diabetes

**DOI:** 10.1186/1758-5996-7-S1-A63

**Published:** 2015-11-11

**Authors:** Ticiana Paes Batista da Silva, Luiz Clemente Rolim, Celso Ferreira de Camargo Sallum Filho, Livia M Zimmermann, Fernando Malerbi, Sergio Atala Dib

**Affiliations:** 1 Universidade Federal de São Paulo (UNIFESP_EPM), São Paulo, Brazil

## Background

Hypoglycemia unawareness affects approximately 25% of patients with type 1 diabetes and is strong associated with severe hypoglycemia. Cardiovascular Autonomic Neuropathy (CAN) is one important factor related to hypoglycemia unawareness, although its role is not fully understood due to conflicting data in the literature. Heart rate variability (HRV) in time and frequency domain has been described as a more accurate method to assess CAN. The objective of the present study was to investigate the relationship between hypoglycemia unawareness and autonomic dysfunction assessed by HRV in time and frequency domain.

## Materials and methods

Type 1 Diabetes patients were prospectively enrolled. Hypoglycemia unawareness was evaluated by Pedersen-Bjegaard method and patients were classified into three groups: normal hypoglycemia awareness (NA), impaired hypoglycemia awareness (IA) and hypoglycemia unawareness (UNA). Indices of HRV were evaluated in both time and frequency domain. CAN was diagnosed using cardiovascular reflex tests (Ewing Battery).

## Results

The study population comprises 99 patients with a mean age of 25.8±10.9 yrs., mean diabetes duration of 12.8±8 yrs. and mean HbA1c 8.4±1.3% (68±10.4 mmol/mol). There was a progressive increase in age (p=0.001), diabetes duration (p=0.027) and episodes of severe hypoglycemia in the last year (p=0.005) as the degree of perception of hypoglycemia decreases. Early CAN was more prevalent in the group UNA (p=0.017). Measures of HRV in frequency and time domain, high frequency band (p=0.027), total power (p=0.037), the square root of the mean squared differences of successive NN intervals (RMSSD) (p=0.041) were progressively decreased with deteriorating hypoglycemia awareness.

## Conclusion

Hypoglycemia awareness impairment in T1D patients is associated with age, duration of disease and the number of severe hypoglycemia episodes in the previous year. Importantly, as hypoglycemia awareness worsens heart rate variability (HRV) decreases denoting a progressive loss of parasympathetic activity on the heart.

